# Supplementary material for: Naphthalene Phthalimide Derivatives as Model Compounds for Electrochromic Materials
Source: Molecules. 2023 Feb 11;28(4):1740. doi: 10.3390/molecules28041740 (PMC9968047; doi:10.3390/molecules28041740)
Supplement: Supplementary file 1 [file molecules-28-01740-s001.zip › molecules-2163716-supplementary.pdf]

Supporting Information

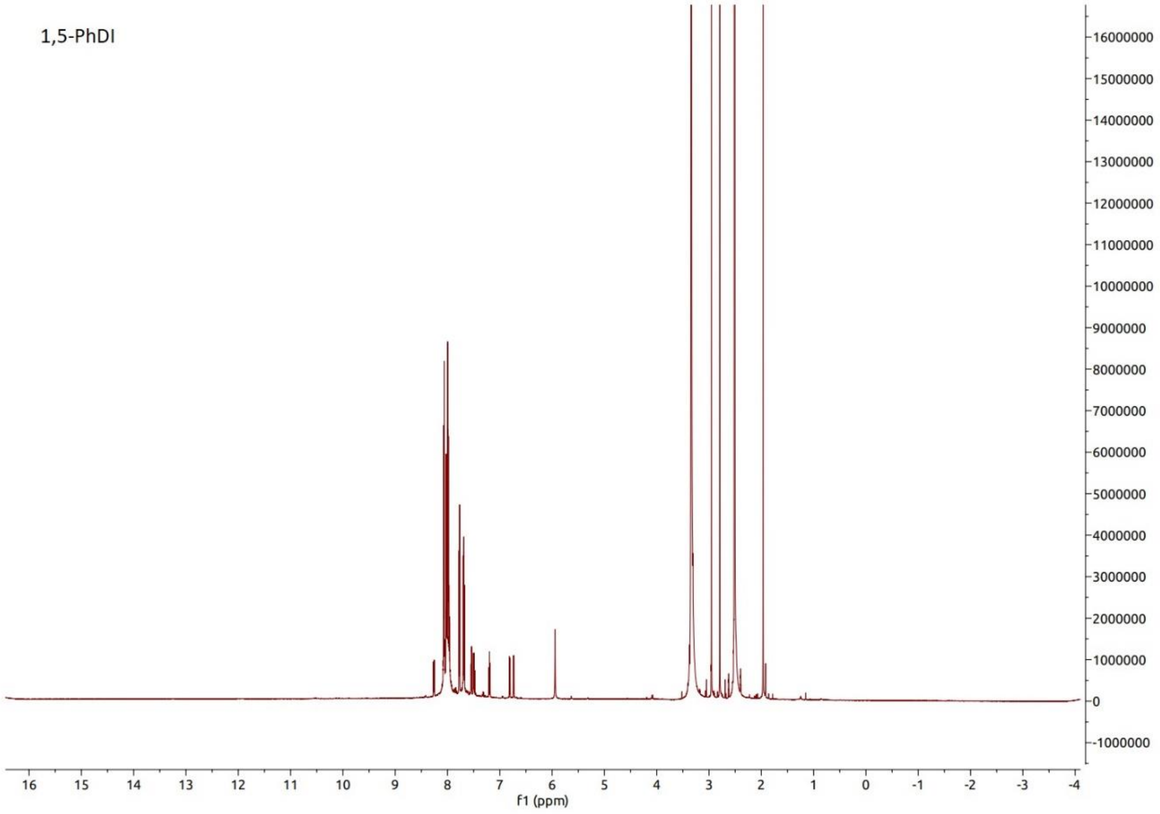

Figure S1. ^{1}H-NMR spectrum of 1,5-PhDI.

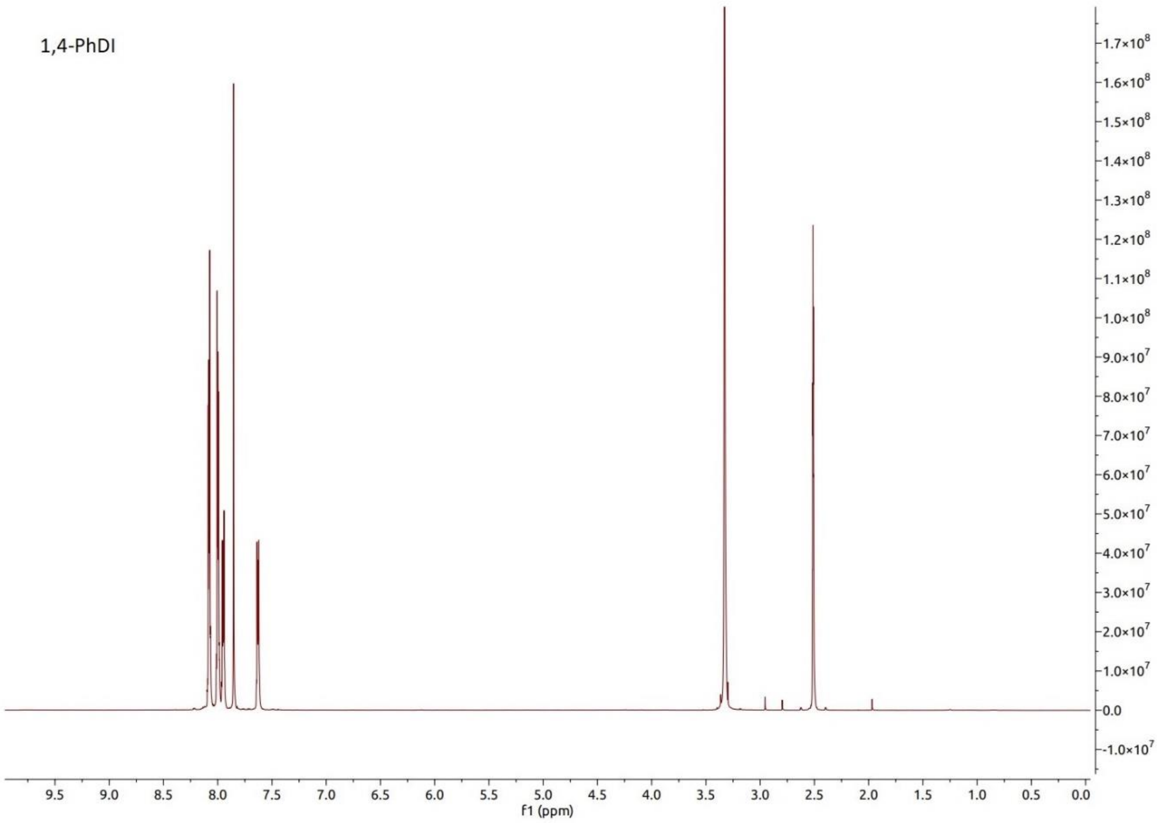

Figure S2. ^{1}H-NMR spectrum of 1,4-PhDI.

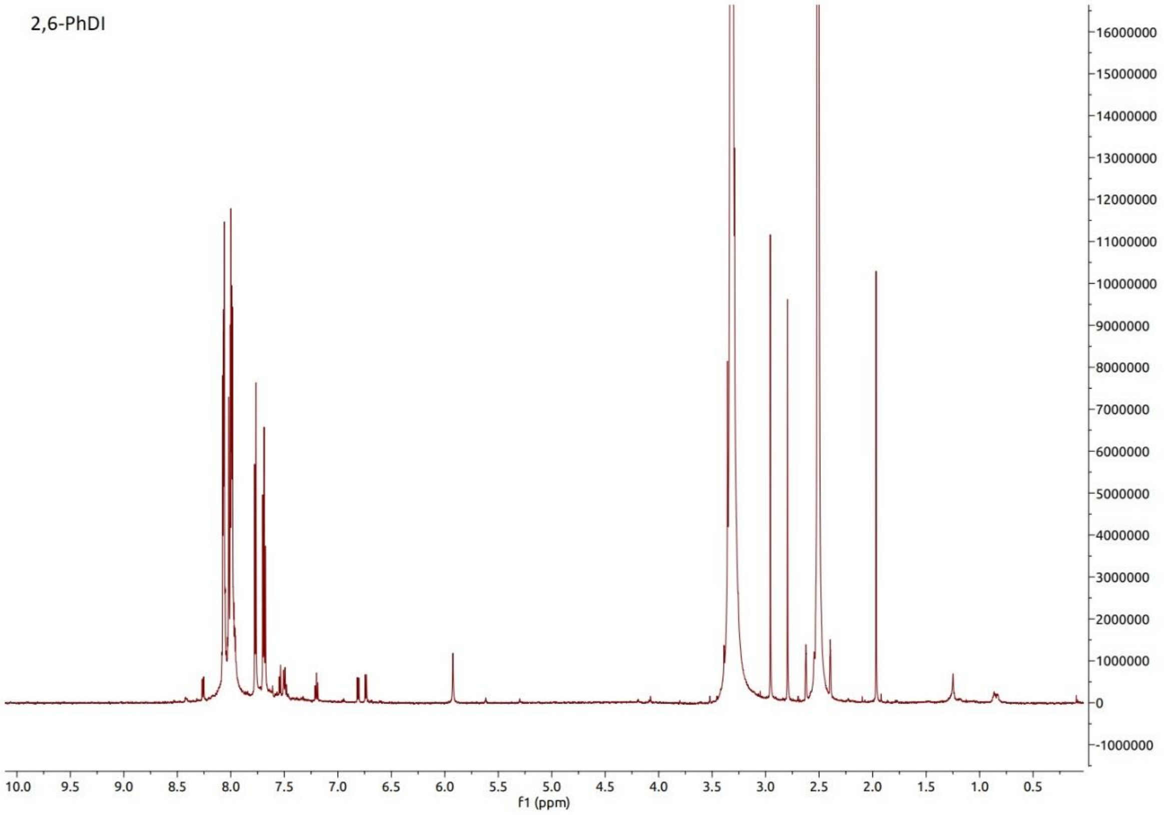

Figure S3. ^{1}H-NMR spectrum of 2,6-PhDI.

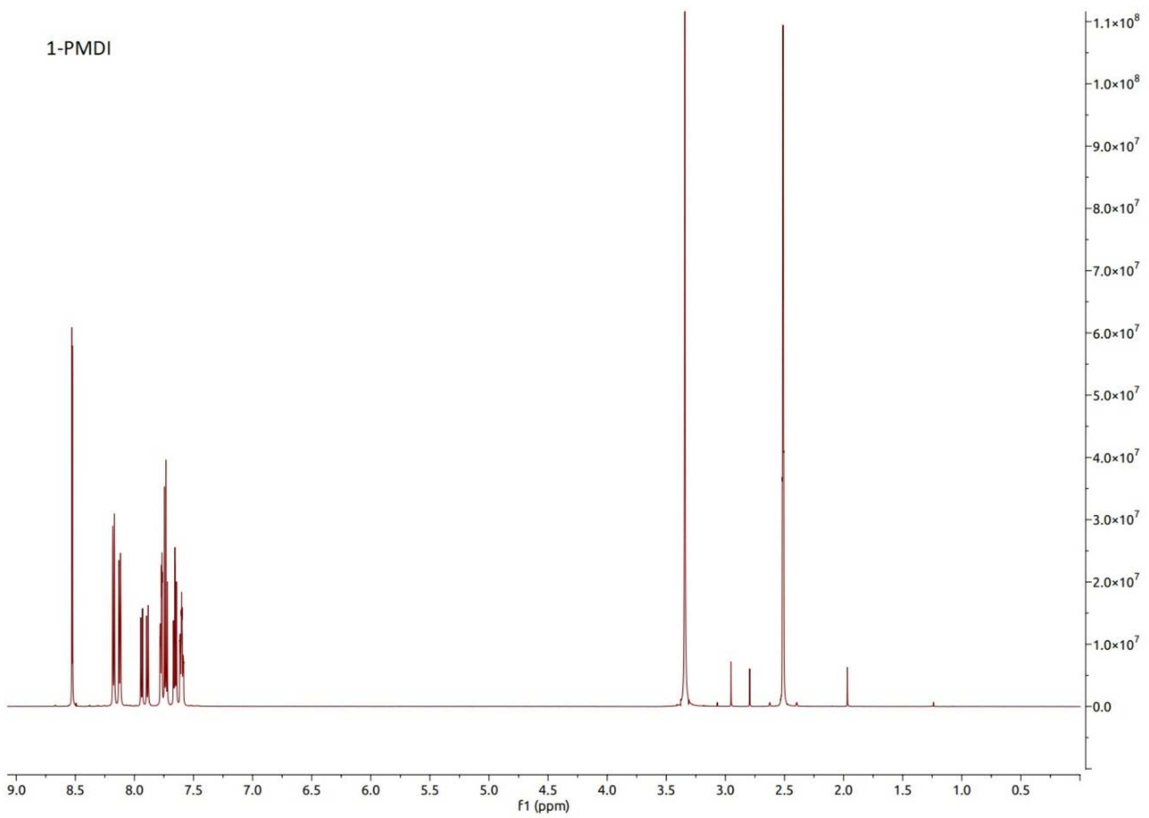

Figure S4. ^{1}H-NMR spectrum of 1-PMDI.

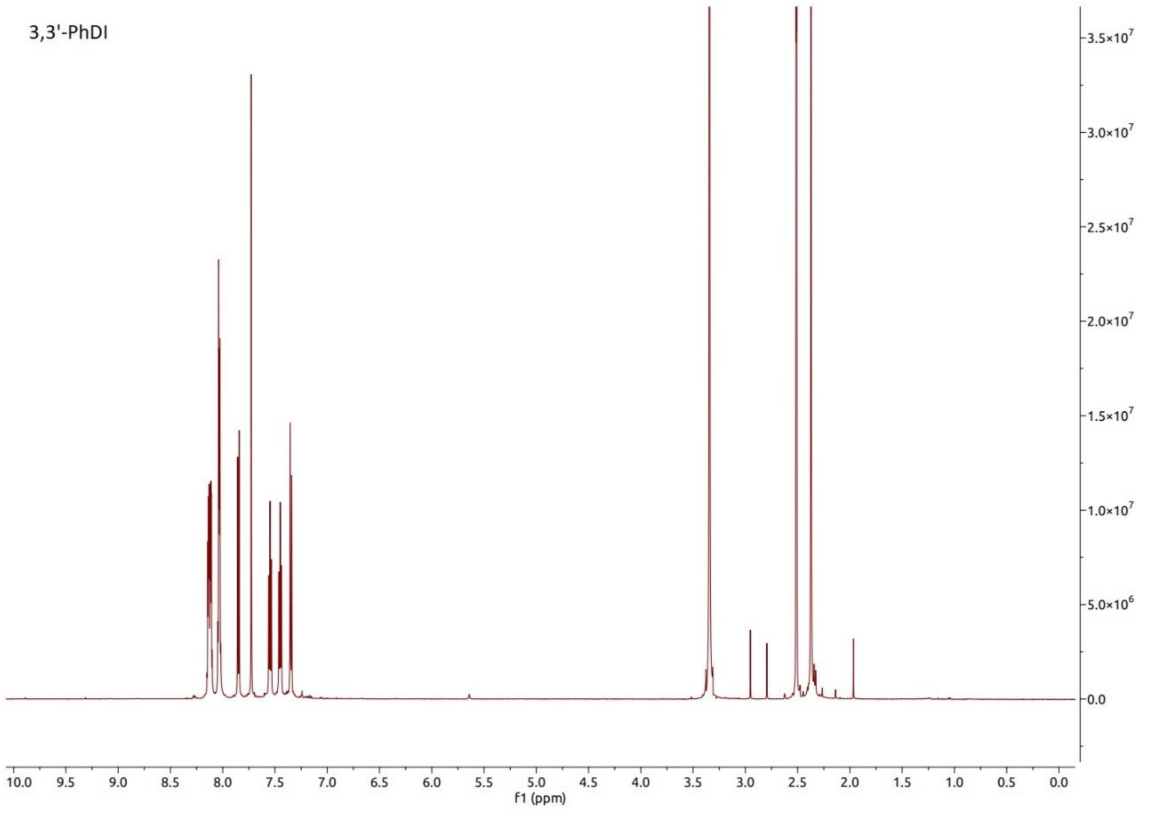

Figure S5. ^{1}H-NMR spectr of 3,3′-PhDI.

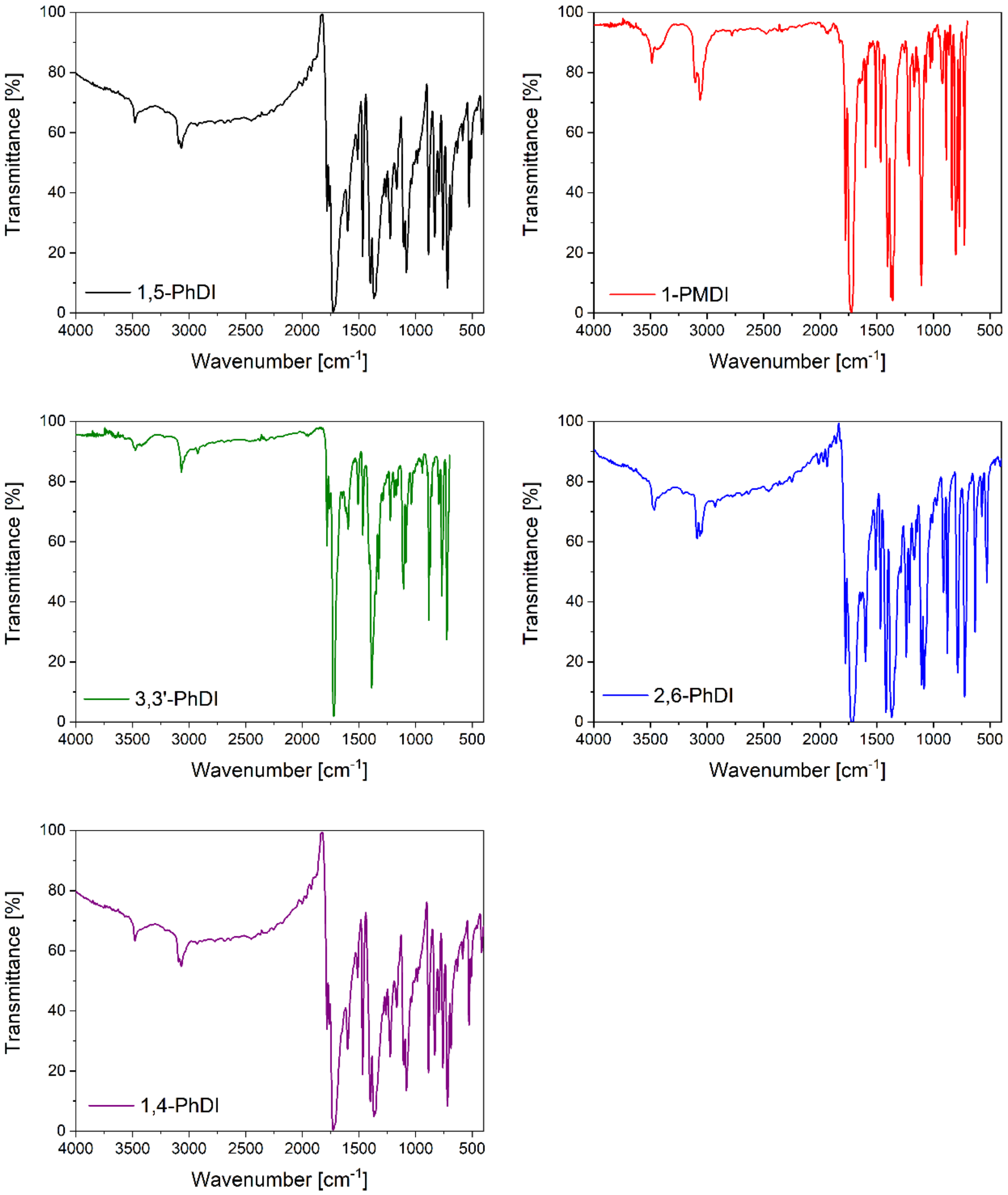

Figure S6. FTIR spectra of investigated compounds.

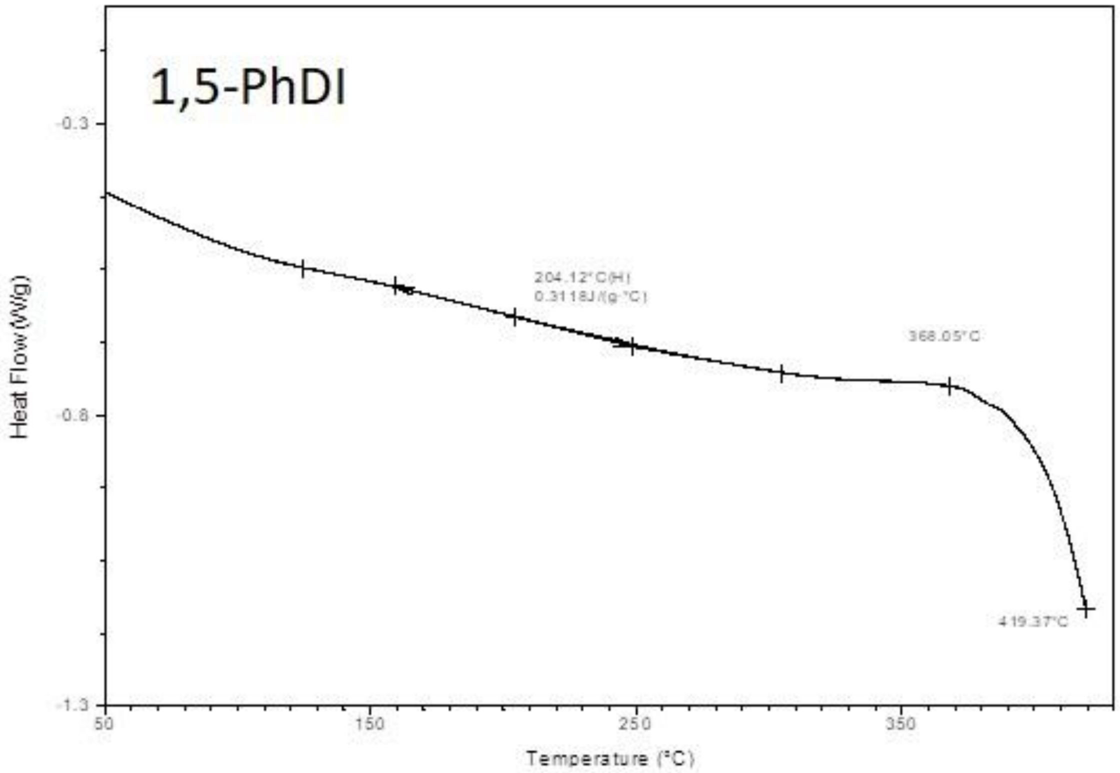

Figure S7. DSC curve registered for 1,5-PhDI during first heating stage.

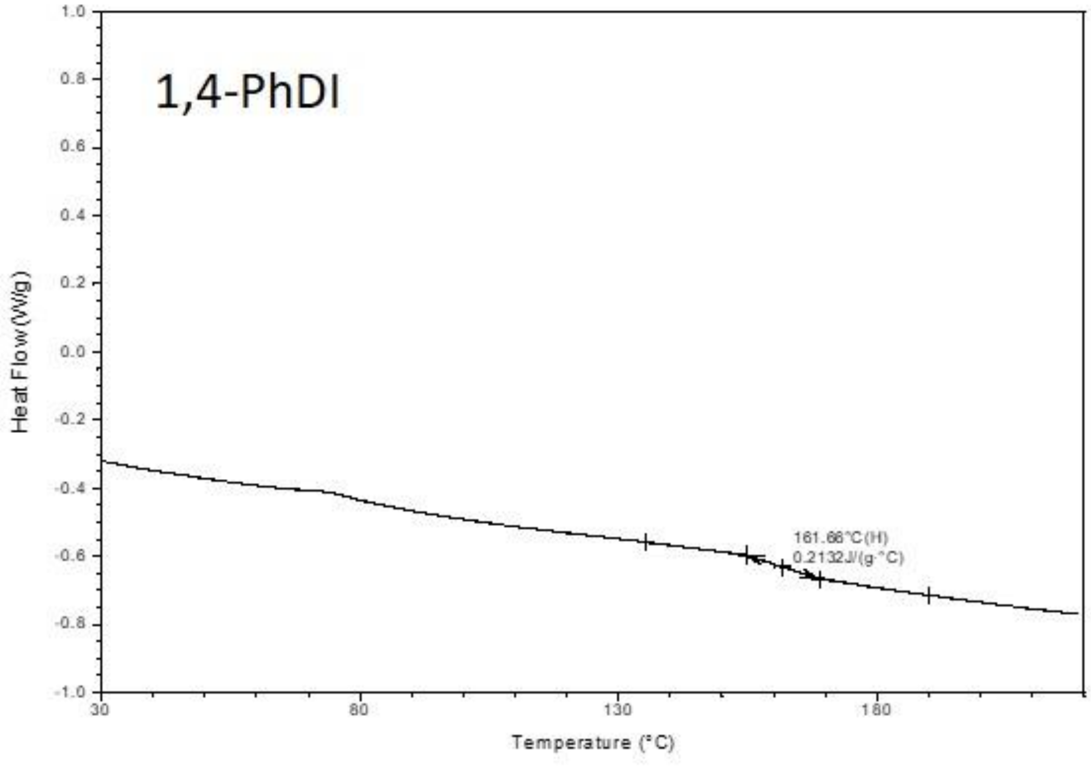

Figure S8. DSC curve registered for 1,4-PhDI during first heating stage.

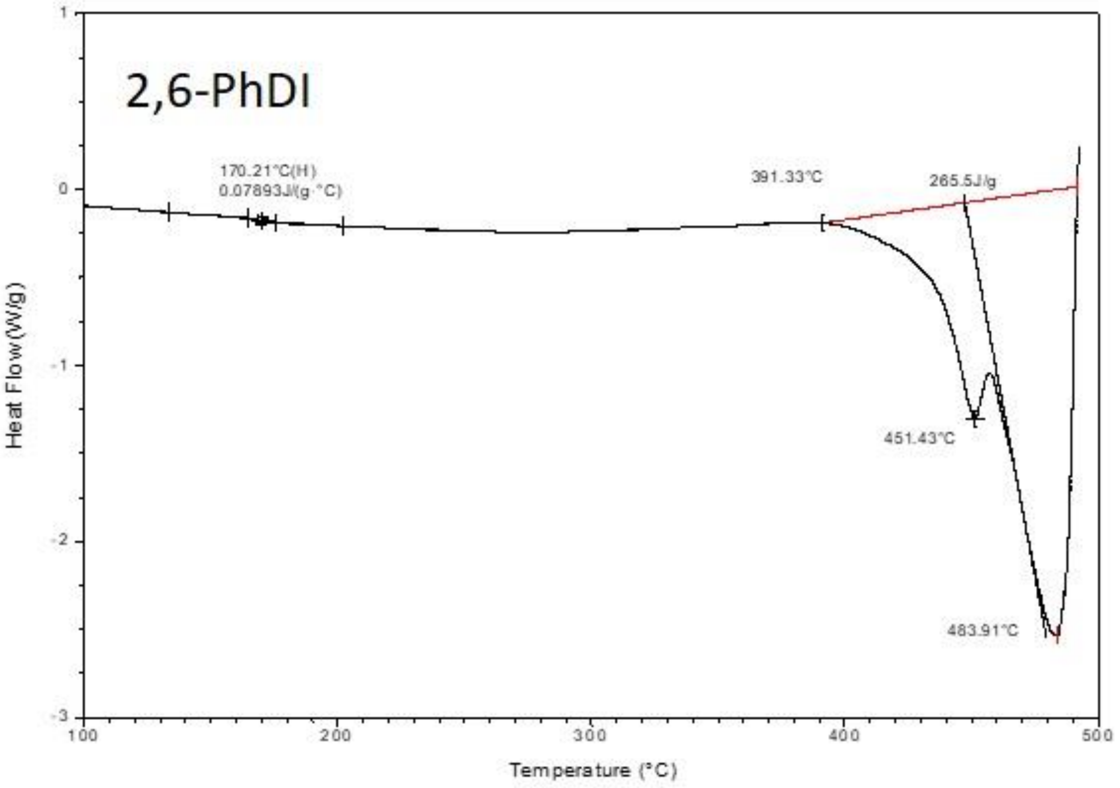

Figure S9. DSC curve registered for 2,6-PhDI during first heating stage.

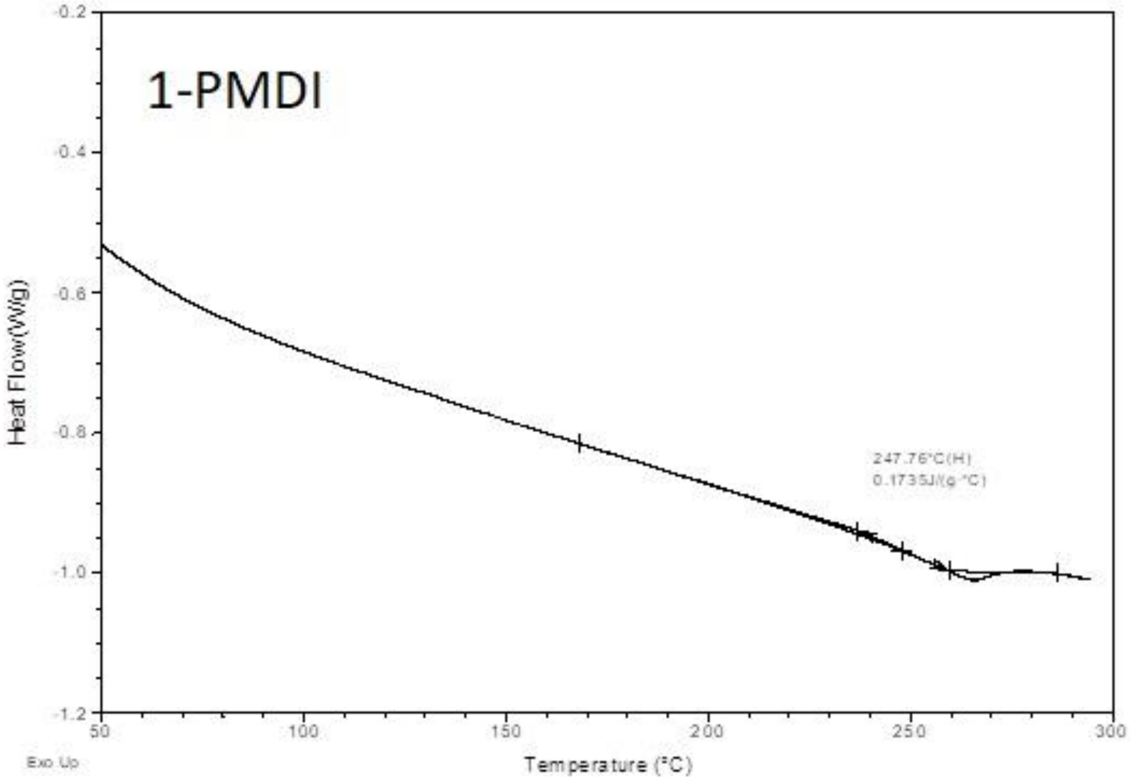

Figure S10. DSC curve registered for 1-PMDI during first heating stage.

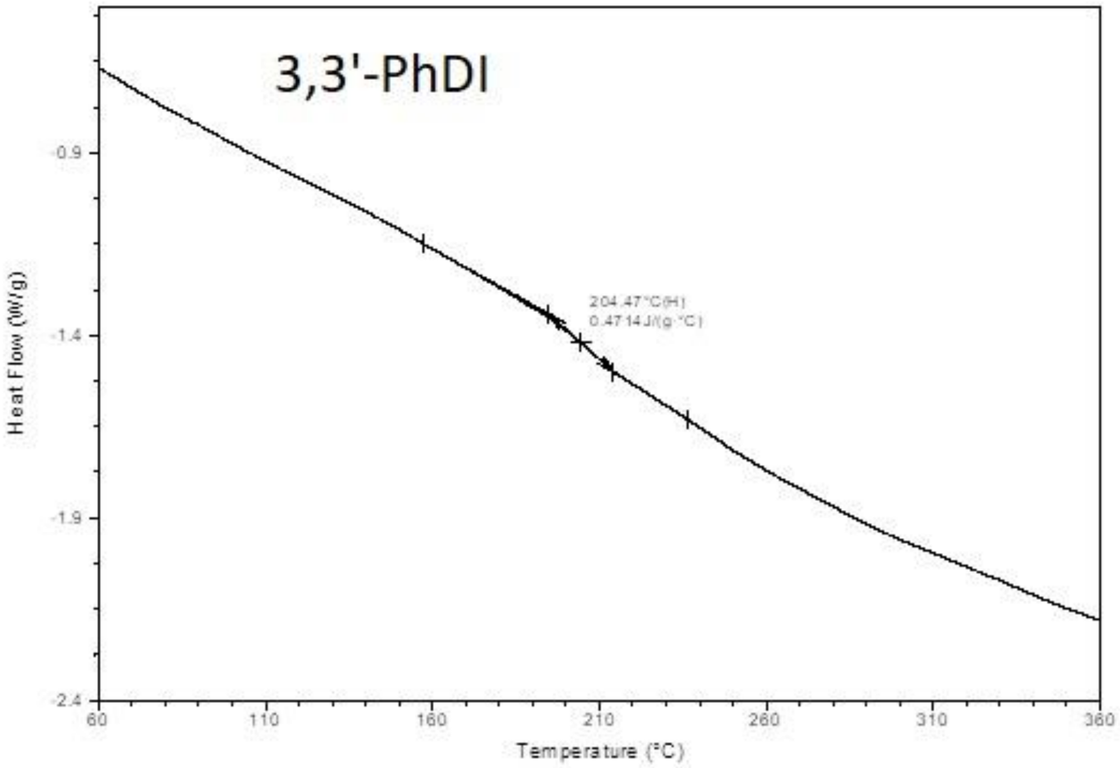

Figure S11. DSC curve registered for 3,3′-PhDI during first heating stage.

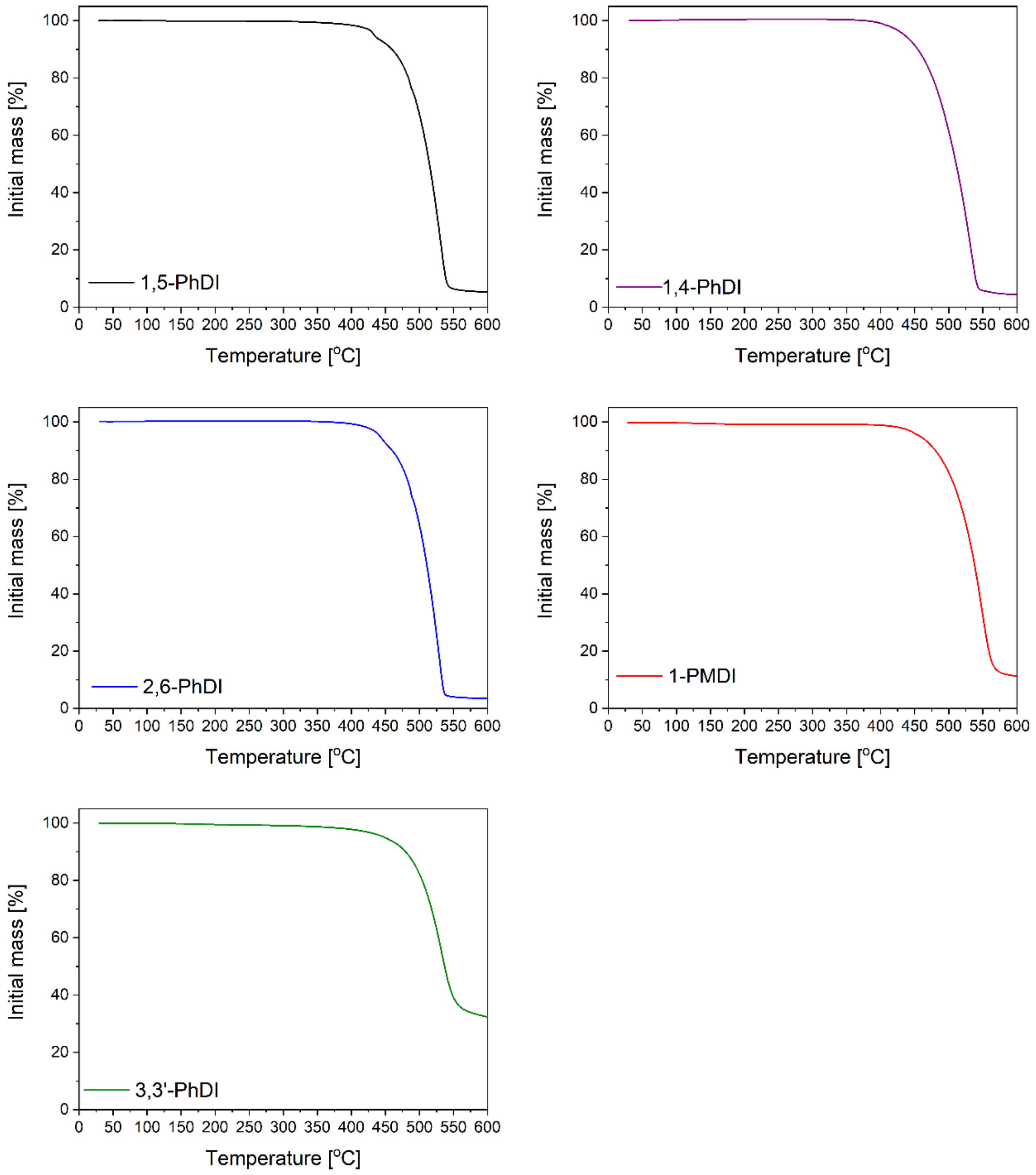

Figure S12. TGA curves registered for investigated compounds.

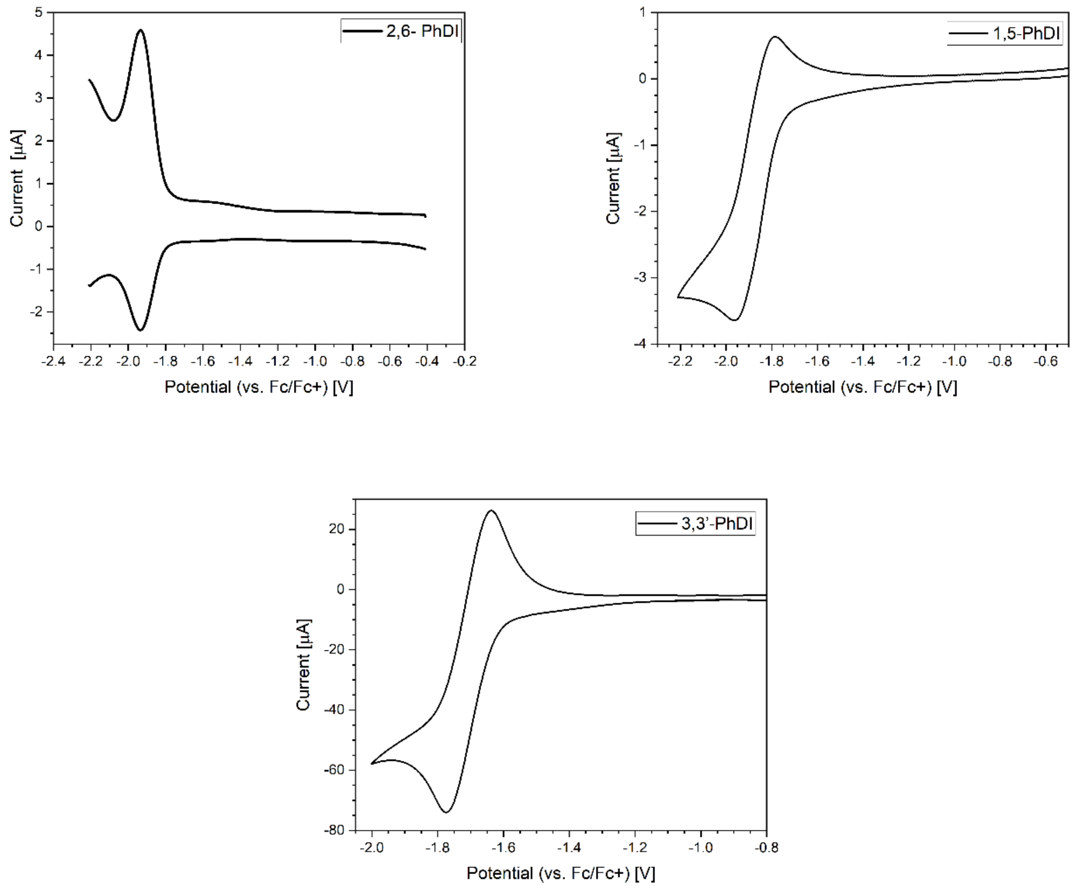

Figure S13 Differential pulse voltammogram of 2,6-PhDI and cyclic voltammograms of 1,5-PhDI and 3,3′-PhDI registered in 0.1 M Bu_4NPF_6/DCM electrolyte; scan rate of 0.1 V/s.

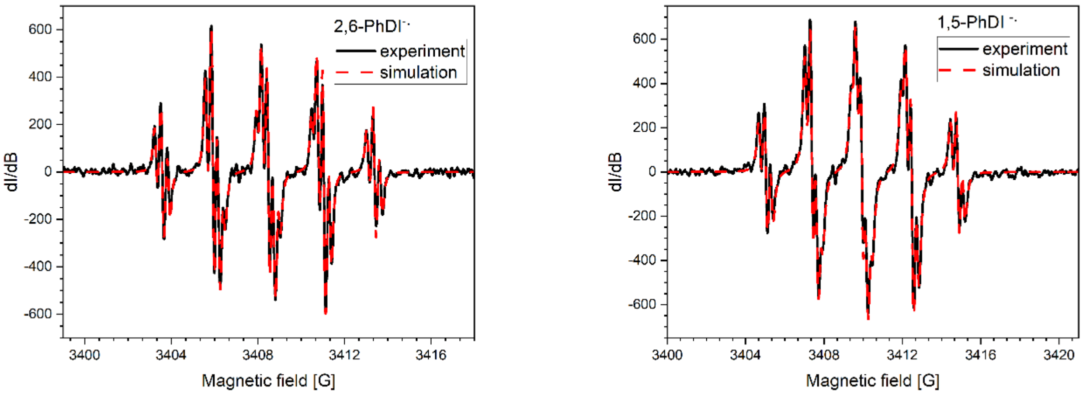

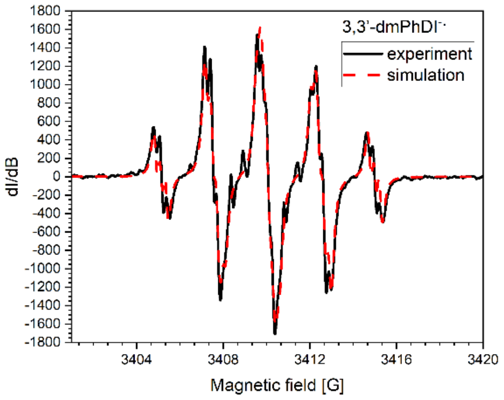

Figure S14. Experimental (black line) and simulated (red dashed line) EPR spectra of electrochemically generated radical anions of 2,6-PhDI, 1,5- PhDI and 3,3′-PhDI in 0.1 M Bu_4NPF_6/DCM electrolyte; fitting parameters are listed in Table 4.

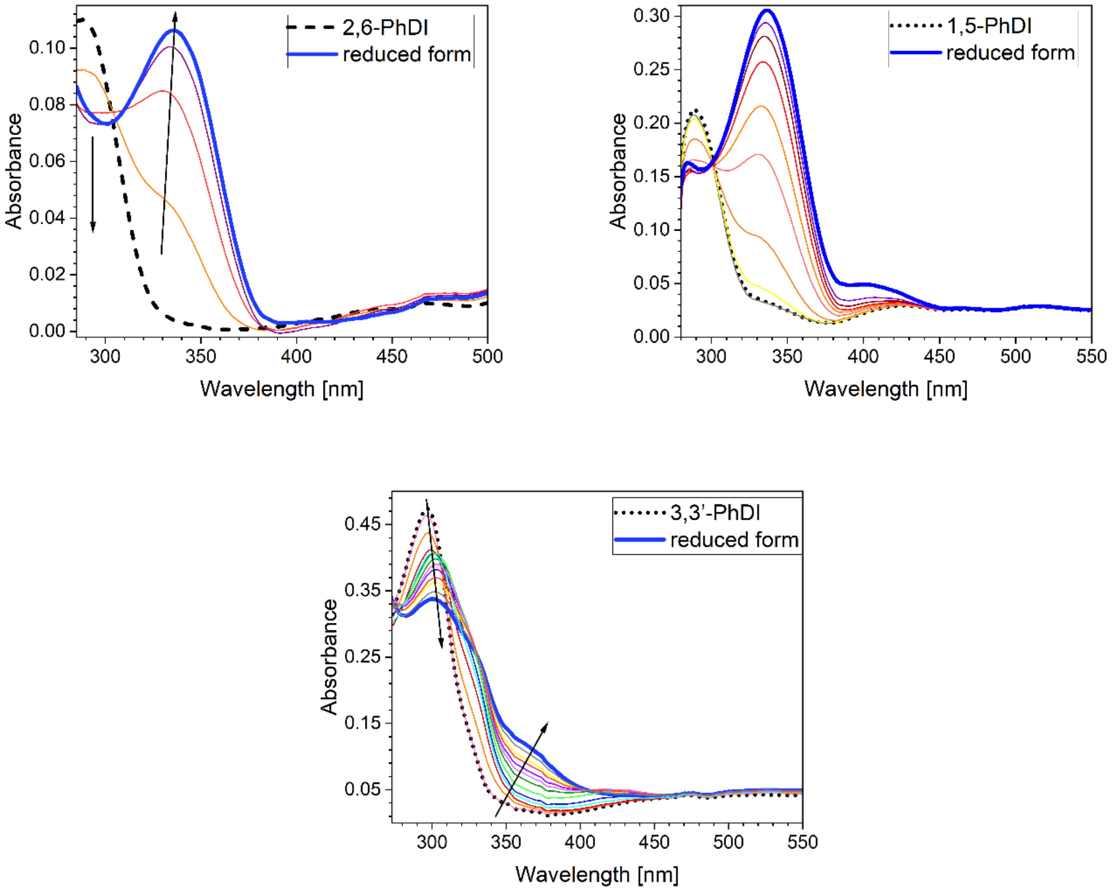

Figure S15. UV-Vis spectra of 2,6-PhDI, 1,5-PhDI and 3.3′-PhDI collected for decreasing working electrode potential in 0.1 M Bu_4NPF_6/DMF electrolyte.

Table S1. Calculated angles between the bridge and imide groups in neutral and reduced form.

Compound	neutral	radicaldianion
1,5-PhDI	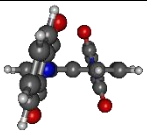	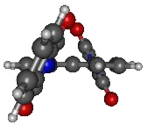
1,4-PhDI	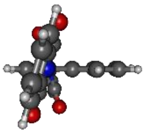	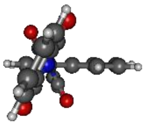
2,6-PhDI	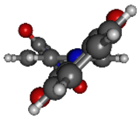	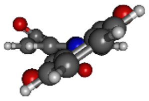
3,3′-PhDI	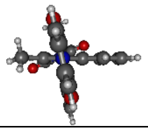	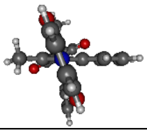
